# Scoring Mating System of a Natural Population of *Neolamarckia cadamba* with Genome Resequencing Data

**DOI:** 10.3390/biology15151262

**Published:** 2026-08-01

**Authors:** Chao Wu, Zi-Yi Gao, Hui Xie, Kun-Xi Ouyang, Qing-Min Que, Xin-Sheng Hu

**Affiliations:** 1 College of Forestry and Landscape Architecture, South China Agricultural University, Guangzhou 510642, China; chaowu1998@163.com (C.W.); ziyigao76916@163.com (Z.-Y.G.); xiehui1721@163.com (H.X.); kxouyang@scau.edu.cn (K.-X.O.); qmque@scau.edu.cn (Q.-M.Q.); 2 Guangdong Key Laboratory for Innovative Development and Utilization of Forest Plant Germplasm, Guangzhou 510642, China

**Keywords:** *Neolamarckia cadamba*, mating system, genome resequencing, MLTR, BORICE

## Abstract

*N. cadamba* is a fast-growing and economically important tree species with significant industrial and medicinal values. Its spherical capitulum comprises hundreds of sessile florets densely aggregated on a receptacle. How this unique floral structure shapes the mating system of this species remains unknown, as this structure enhances the probabilities of both selfing and outcrossing. Here we clarify this question by investigating a natural population. We randomly sampled 11 half-sib families and 6 progenies per half-sib family and assayed samples with whole-genome resequencing. Population genomic analysis indicated that about 93.09% of 4,876,674 high-quality SNPs were under Hardy–Weinberg equilibrium, with a mean inbreeding coefficient of 0.0614 in the sample. Genome-wide nucleotide diversity (π) was 0.0024 on average. The linkage disequilibria (r^2^) decayed by half at approximately 2.31 kb between sites across genomes. Principal component analysis showed clear family structure among all samples. Analysis with independent SNPs and MLTR (Program for estimating multilocus outcrossing rate) showed that the multilocus outcrossing rate (*t_m_*) and single-locus outcrossing rate (*t_s_*) were 1.028 ± 0.048 and 1.015 ± 0.060, respectively, with an estimated difference between *t_m_* and *t_s_* of 0.013 ± 0.022. Analysis with BORICE (Bayesian outcrossing rate and inbreeding coefficient estimation) showed an outcrossing rate (*t*) of 0.948 ± 0.029 and an inbreeding coefficient (*F*) of 0.012 ± 0.010. Collectively, these results imply that *N. cadamba* could be essentially outcrossing, without selfing and biparental inbreeding. The potentially outcrossing system of *N. cadamba* implies differences from its close relative *N. macrophylla* (a mixed-mating system) in population structure, genome evolution, and natural geographical distribution. This finding also provided a useful reference for breeding and genetic improvement of this “miracle tree”.

## Simple Summary

A mating system as a life history trait describes how genetic material is transmitted across successive generations. It can interact with basic evolutionary forces to shape population structure and speciation. Relevant empirical studies have advanced from the previous use of molecular markers to the current use of genome-wide SNPs (single nucleotide polymorphisms). Here we used genome re-sequencing data to study the mating system of *Neolamarckia cadamba*, a fast-growing tropical tree with significant industry and medicinal values. Its hermaphroditic flowers are densely packed into a spherical head inflorescence, and pollination is primarily mediated through various insects (bees, butterflies, and beetles) due to nectar production. We investigated a natural population and conducted population genomic analysis. Genomic evidence supported that the trees exhibited high genetic diversity and very low levels of inbreeding. The natural population was essentially outcrossing, in contrast to the mating system of its close relative *N. macrophylla* (a mixed mating system). This finding helps us to infer the ecological and evolutionary divergences between *N. cadamba* and *N. macrophylla*. It also provides a valuable reference for breeders to design appropriate breeding programs and to develop a genetic conservation strategy for this species.

## 1. Introduction

*N. cadamba* belongs to the *Neolamarckia* genus of the Rubiaceae family. This genus comprises only two species (*N. macrophylla* is the other species) that form a monophyletic group [1,2]. *N. cadamba* is naturally distributed across regions with favorable hydrothermal conditions, including South China, Southeast Asia, South Asia, and Northern Australia. In contrast, *N. macrophylla* is naturally distributed only on the Sulawesi and Maluku Islands, Indonesia, and is a threatened species. The two species coexist on the two islands [3,4] and diverged more recently [5]. Two features are notable for these two species in the Rubiaceae family. One is that *N. cadamba* has been applied to traditional medicine for a long history in Southeast and South Asian countries. Its bark extracts have been used to treat fever, cough, and diabetes. Modern studies have demonstrated that the bark extracts were rich in cadambine, various flavonoids, and terpenoid compounds, exhibiting significant pharmacological activities, including antioxidant, antidiarrheal, and antitumor effects [6,7,8]. The other feature is its rapid growth and relatively delayed senescence, with an age of reproductive maturity at approximately eight years. The tree can reach up to 45 m in height, and the trunk diameter can attain 1.6 m. The wood is of high quality, possesses considerable economic value, and is easy to process, making it suitable for furniture, construction, decorative materials, and pulp production [9,10,11]. In addition, the species is also planted in wetlands for ecological restoration. Owing to these multiple uses, *N. cadamba* was often referred to as a “miracle tree” and regarded as a resource species with substantial development potential and broad application prospects [7,11,12].

**Figure 1:**
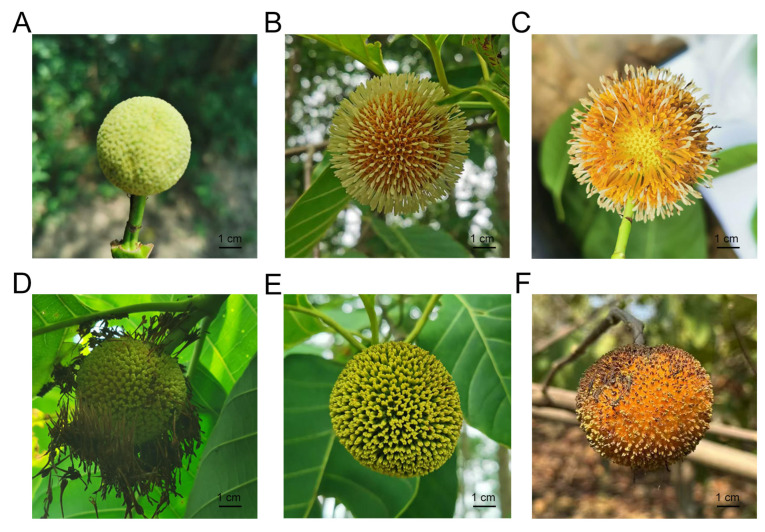
The process of flowering and fruiting of *N. cadamba*. (**A**) Bud stage; (**B**) blooming; (**C**) corolla senescence; (**D**) flower shedding; (**E**) fruit development; and (**F**) fruit maturation. These figures were taken by CW.

The inflorescence of *N. cadamba* is a globose capitulum borne terminally on branches. The peduncle is 2–4 cm long, with hundreds of sessile flowers densely arranged on an enlarged receptacle; at anthesis, the inflorescence reaches 40–60 mm in diameter. Individual flowers are bisexual and homomorphic, with a five-lobed calyx and a yellow to white funnel-shaped corolla possessing a relatively long tube (~10 mm). Both corolla lobes and stamens are five; the stamens are inserted near the upper part of the corolla tube and are partially exserted. Filaments are short, and anthers are basally attached. Seeds are minute (0.5–0.7 mm in length) and produced in large quantities [4]. The globose capitulum of *N. cadamba* was regarded as a highly integrated reproductive unit, in which numerous bisexual flowers were densely arranged on an enlarged receptacle, forming a compact spatial structure (Figure 1). The structural integration of these flowers could exert a significant impact on pollination of the capitulum. Since a large number of flowers within the same inflorescence are highly proximate in space and bloom almost synchronously, this arrangement potentially facilitates geitonogamy, thereby increasing the likelihood of self-fertilization. Conversely, as these pseudanthia are entomophilous, the synchronous flowering of numerous florets combined with abundant nectar secretion effectively attracts pollinators. This enhances pollen transfer among different individuals, thus promoting outcrossing. Nevertheless, the mating system of this species, an important life history trait, remains unclear.

A previous study with SSR markers indicated that *N. macrophylla* possessed an average outcrossing rate of 76.9% and a selfing rate of 23.1% in three natural populations, and pollen dispersal distances could reach up to 339 m, suggesting a mixed-mating system with an outcrossing preference [13]. Although the mating system of *N. cadamba* has not been scored, two relevant findings imply that it potentially has a mating pattern with a comparable or higher outcrossing rate than *N. macrophylla*. The first finding is that population genetic variation across the whole range of natural distribution in China was mainly distributed within populations (*F_st_* ≈ 3% for the nrDNA ITS markers). Gene flow was mainly achieved through pollen flow rather than seed flow despite the fact that pollination is mediated by insects [14]. The second finding is about genome heterozygosity. The genome of *N. cadamba* was sequenced and assembled in 2022, with a total size of approximately 744.5 Mb, a repeat content of 54.29%, and a genome-wide heterozygosity of 0.69% [15]. Previous studies demonstrated that the mating system was one of the key drivers of genome evolution, influencing genome-wide heterozygosity through its effects on effective population size (*N_e_*), recombination rate, and homozygosity. Long-term selfing resulted in originally heterozygous loci rapidly approaching homozygosity, thereby significantly reducing genome-wide heterozygosity; in contrast, outcrossing promoted allelic recombination and reshuffling, effectively maintaining relatively high levels of heterozygosity [16,17,18]. These were supported by studies on *Oryza nivara* and *O. rufipogon* [19], *Primula* species [20], and *Juniperus osteosperma* [21]. Compared with these species, the 0.69% genomic heterozygosity in *N. cadamba* represents a substantially high level. Given a close genetic relationship between the two species in the genus *Neolamarckia* [1,5], it is hypothesized that *N. cadamba* is predominantly outcrossing, potentially accompanied by low levels of selfing or biparental inbreeding.

Knowledge of plant mating systems helps us to infer genetic variation within and between populations, such as genotypic composition, population genetic differentiation, and linkage disequilibrium between loci. Scoring the mating system of *N. cadamba* is of practical significance for genetic breeding and evaluating progeny performance where seeds are collected from natural or breeding populations. Our current ongoing breeding programs are based on open-pollinated seeds derived from natural populations with an unknown mating system [22]. The presence of selfing or inbreeding could yield biased estimates of genetic parameters of important quantitative traits under the assumption of random mating. In addition, this knowledge gap limits our understanding of how its highly integrated inflorescence structure shapes ecological and evolutionary consequences of this species. Therefore, elucidation of the mating system of *N. cadamba* helps us to gain insights into how such capitulum architecture affects reproductive patterns and ecological and evolutionary processes.

With the advancement of high-throughput sequencing technologies, studies on plant mating systems have advanced from conventional field surveys and theoretical modeling to the use of isozyme markers [23], SSR (microsatellite) markers [24,25], and genome-wide SNPs. Compared with previous molecular markers, genome-wide SNP markers offered several advantages, including extremely high distribution density, strong stability, and genome-wide coverage. These attributes enable more precise estimation of mating system parameters, such as selfing and outcrossing rates [26,27]. Currently, software such as MLTR and BORICE is a useful tool for this purpose [28,29]. Genomic evidence on how this capitulum shapes the trade-off between selfing and outcrossing remains scarce. The inflorescence architecture of *N. cadamba* provides an ideal system for dissecting this relationship. It is expected that the mating system of *N. cadamba* can be more accurately assessed at the genomic level.

In this study, we utilized whole-genome resequencing data from 11 half-sib families (66 individuals in total) randomly sampled from a natural population of *N. cadamba*. Through estimating the selfing rate, biparental inbreeding coefficient, and outcrossing rate, we assessed the mating system of *N. cadamba* and inferred the potential effects of its highly integrated inflorescence architecture on reproductive strategy. The results would provide a genetic basis for the efficient utilization of germplasm resources and the genetic improvement of this species.

## 2. Materials and Methods

### 2.1. Plant Materials and Genome Re-Sequencing

A natural population in Jinghong city (100.81° E, 22.03° N, 552 m above sea level), Yunnan Province, was selected. This population had the most half-sib families among 11 provenances in a provenance-progeny trial, which was established at the Jijia Town Forest Farm in Leizhou (21°10′06″ N, 110°21′34″ E), Guangdong Province, in 2014. Seed samples for the provenance-progeny trial were collected from mother trees that were separated with a minimum distance of 100 m in the same forest stand so as to avoid genetic relatedness. Jinghong population is located in the regions with the most abundant natural distribution of *N. cadamba* in China and could be a representative sample of natural forests. Seeds from 11 randomly selected mother trees were collected in the Jinghong population, and their seedlings, together with seedlings of the remaining 10 provenances, were planted in this trial [22]. The climatic conditions of the experimental site belong to the tropical humid monsoon climate, with an average annual sunshine duration of 2031.5 h, a mean annual temperature of 23.6 °C, and an average annual precipitation of approximately 1671.5 mm, providing an ideal environment for the growth of *N. cadamba*. A completely randomized block design was employed for the experiment, with a planting density of 3 m × 3 m. Six healthy leaf samples for each half-sib family (66 individuals in total) were collected for whole-genome resequencing (Table 1). Note that the sample sizes (6 seeds per half-sib family) are appropriate when multiple or highly polymorphic markers are employed [25]. Previous simulation studies indicated that the optimal number of progeny per family was from 4 to 8 zygotes for estimating fixation index and selfing parameters [30].

The whole-genome resequencing data came from two public repositories. Specifically, forty-four samples of resequencing data were retrieved from the China National GeneBank (CNGB) Nucleotide Sequence Archive (CNSA) (https://db.cngb.org/) under the project accession number CNP0007971 (https://db.cngb.org/data_resources/project/CNP0007971, accessed on 29 June 2026) and coded by C-series in Table 1. The remaining twenty-two samples were obtained from the NCBI BioSample database (accession number: SAMN15700859) (https://www.ncbi.nlm.nih.gov/biosample/SAMN15700859, accessed on 20 September 2025), which originated from Zhao et al. [15] and were coded by D-series in Table 1. For all samples, re-sequencing was performed following the standard procedures provided by Illumina, including DNA quality control, library construction, library quantification, and sequencing (see Supplementary Materials File S1 for detailed protocols). Finally, the VCF files from these two sources were merged using bcftools v1.4 [31].

### 2.2. Population Genomic Analysis

#### 2.2.1. Genome-Wide SNP Distribution

Strict quality control was performed on the resequencing data of the 66 individuals using VCFtools (v0.1.16) [32]. Scaffold sequences were excluded to retain only loci on 22 chromosomes. Further filtering was applied to yield a high-quality dataset of bi-allelic SNPs by retaining loci with exactly two alleles (--min-alleles 2 --max-alleles 2), a minor allele frequency (MAF) ≥ 0.05, and a locus missing rate ≤ 20%. The R package CMplot (v4.5.1) was employed to visualize the genome-wide distribution of SNP density (https://github.com/YinLiLin/CMplot, accessed on 1 March 2026). Specifically, the SNP density was evaluated by calculating the number of SNPs within a 100 kb window size.

#### 2.2.2. Hardy–Weinberg Equilibrium

Hardy–Weinberg equilibrium (HWE) was tested for each SNP using VCFtools with the --hardy parameter. The test compared the observed genotype frequencies at each SNP locus with those expected under HWE. The degree of deviation was evaluated using an exact test, and the resulting *p*-values were adjusted by using the false discovery rate (FDR) method (Benjamini–Hochberg) in R, with the statistical significance set at FDR < 0.05. The distribution of *p*-values was then visualized as a histogram using Matplotlib v3.10.6 [33] to assess the overall genomic adherence to HWE.

#### 2.2.3. Estimation of Inbreeding Coefficients ($F_{is}$)

The observed heterozygosity (*H_o_*) and expected heterozygosity (*H_e_*) under HWE were calculated for each SNP locus in R (v4.4.3) [34]. Inbreeding coefficients (*F_is_*) at individual SNP sites were estimated according to $F_{is}=1-H_{o}/H_{e}$. To assess the robustness of these parameter estimations, a non-parametric Bootstrap approach with 1000 iterations of locus-based resampling with replacement was implemented to construct the 95% confidence intervals (CI) for $F_{is}$. The distribution of $F_{is}$-values was visualized in a histogram plot using Matplotlib [33].

#### 2.2.4. Genetic Diversity

The genome-wide nucleotide diversity (π) was calculated using the sliding window method by VCFtools (v0.1.16) [32], with a window size of 10 kb and a step size of 1 kb (--window-pi 10000, --window-pi-step 1000). The density distribution of π values was calculated at 1 kb intervals, and the results were visualized using R v4.4.3 [34].

#### 2.2.5. Linkage Disequilibrium

Pattern of linkage disequilibrium (LD) decay with physical distance between SNPs was calculated using PopLDdecay (v1.90). LDs were plotted, with the physical distance (kb) on the *x*-axis and the standardized LD coefficient (r^2^) on the *y*-axis [35].

#### 2.2.6. Principal Component Analysis (PCA)

To investigate the population genetic structure and family relatedness while minimizing the impact of LD on downstream analyses, the SNP dataset was pruned using PLINK v1.90b6.20 with the parameter (--indep-pairwise 50 10 0.2), and a set of approximately independent SNPs was extracted for subsequent analyses [36]. PLINK was then used to calculate the genetic relationship matrix (GRM) among the 66 individuals. Subsequently, eigen decomposition of the GRM was performed in R using the eigen() function to determine the proportion of total genetic variance explained by each principal component. Finally, the distribution of samples was visualized by the first two principal components (PC1 and PC2) using the ggplot2 v4.0.1 package in R to assess population genetic stratification and family-level relatedness.

### 2.3. Scoring Mating System

#### 2.3.1. MLTR Analysis

To minimize the biases from Hardy–Weinberg disequilibrium (HWD) and LD, we removed the loci that significantly deviated from HWE (*p* < 10^−6^) using VCFtools, followed by strict linkage pruning using PLINK with the parameters (--indep-pairwise 50 10 0.1). SNPs under HWE and linkage equilibrium were screened.

The MLTR software (v3.4, revised in 2019) was primarily designed for low-density and independent genetic markers, making it difficult to directly apply the program to analyze high-density, genome-wide resequencing data. To determine the optimal number of SNPs for MLTR, simulations were performed with different numbers of independent SNPs (2, 4, 6, 8, 10, 15, 20, 25, and 30), and thirty random datasets were independently generated for each case. Each generated dataset was subsequently converted into the input format required by the MLTR software. For simplicity, SNPs on chromosome 1 (the longest chromosome) were examined to screen the optimal number of SNPs for analysis. Subsequently, MLTR analyses were performed on each dataset to estimate the multilocus outcrossing rate (*t_m_*) and its standard deviation, thereby assessing the stability of estimates under different SNP sizes.

The MLTR was used to estimate mating system parameters by the expectation-maximization (EM) method, with default parameter settings (*t* = 0.9, *F_M_* = 0.1, *r_t_* = 0.1, *r_p_* = 0.1) and 1000 resampling iterations [28]. Estimates included the multilocus outcrossing rate (*t_m_*), the single-locus outcrossing rate (*t_s_*), the difference between *t_m_* and *t_s_*, the single-locus inbreeding coefficient (*F*) of maternal parents, the correlation of selfing (*r_s_*) among families, the multilocus correlation of paternity (*r_p_*_(*m*)_), the single-locus correlation of paternity (*r_p_*_(*s*)_), the difference between *r_p_*_(*s*)_ and *r_p_*_(*m*)_, and the correlation of selfing among loci (*r_loci_*). The distribution of these parameters was visualized using matplotlib [34].

#### 2.3.2. BORICE Analysis

The BORICE (v1.1) employs a Bayesian method to jointly estimate the population outcrossing rate (*t*) and inbreeding coefficient (*F*) [29]. To determine the optimal number of SNPs, BORICE adopted the sampling strategy identical to that used for MLTR. Specifically, SNP subsets were randomly sampled from chromosome 1 across a gradient of designated quantities (2, 4, 6, 8, 10, 15, 20, 25, and 30 SNPs) and converted into the input format required by BORICE. Subsequently, BORICE analyses were performed on each SNP dataset to estimate the outcrossing rate (*t*) and its standard deviation (SD), thereby assessing the stability of estimates under different SNP sampling sizes. The analysis used the following parameters: 10,000 steps, 3000 burning steps, an outcrossing rate tuning parameter of 0.05, an allele frequency tuning parameter of 0.1, and an initial population outcrossing rate of 0.5. The distribution of *t* and *F* values was visualized using matplotlib [34].

## 3. Results

### 3.1. Population Genomic Structure

**Figure 2:**
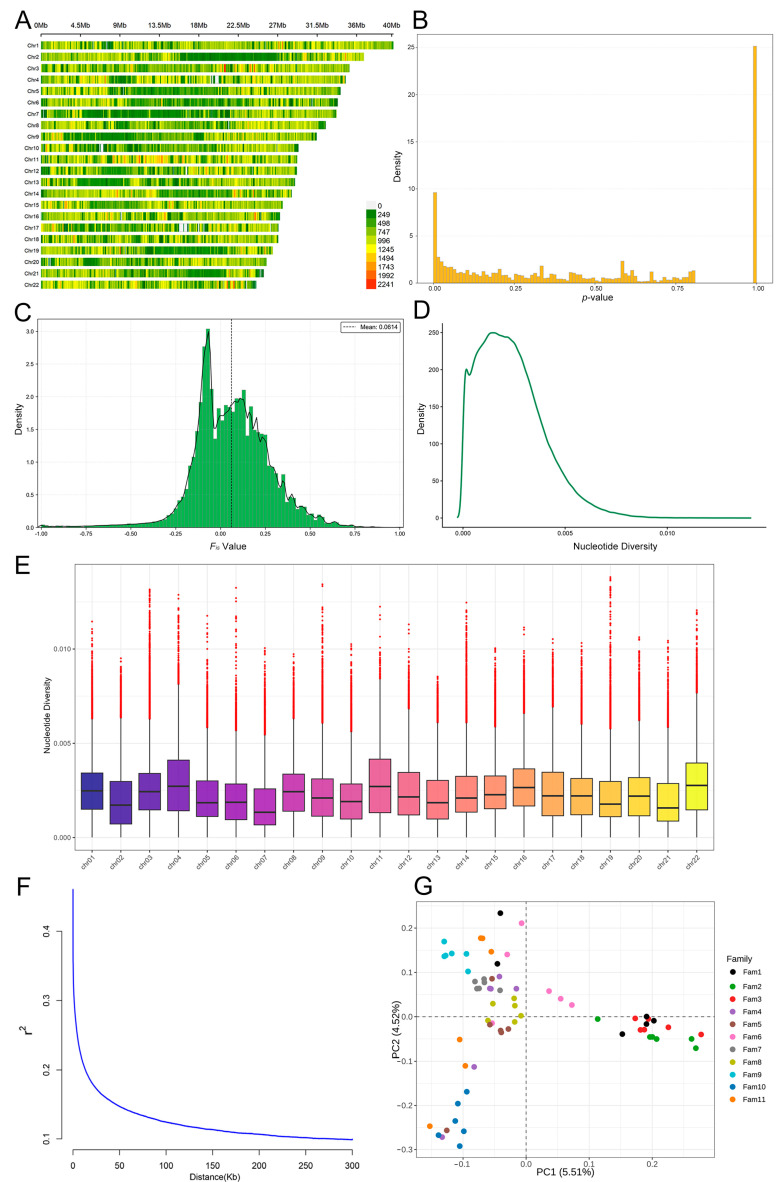
Population genomic variation: (**A**) Genome-wide SNP density visualized in 100 kb window sizes. (**B**) Distribution of *p*-values for HWE tests across 4,876,674 SNPs; (**C**) distribution of *F_is_* estimates across 4,876,674 SNPs; (**D**) distribution of π calculated in 1 kb sliding windows along the genome (4,876,674 SNPs); (**E**) distribution of π calculated across all chromosomes (4,876,674 SNPs) that are indicated in boxes in different colors. The outliers of π values are indicated in red for each chromosome; (**F**) LD decaying pattern (4,876,674 SNPs); (**G**) PCA with 345,349 SNPs.

After strict quality control, a total of 4,876,674 high-quality SNPs from an initial 11,837,583 SNPs were retained for downstream analyses. To assess the genomic distribution of SNPs, we used the CMplot package in R to visualize SNP density within a 100 kb window size, revealing sequencing coverage across genomes (Figure 2A). Chromosome 1 contained the most abundant SNPs (321,784, accounting for 6.60% of the total), while chromosome 21 contained the fewest (167,785, 3.44%). As expected, the SNP counts were positively correlated with the chromosome length. The average density across chromosomes was 138.45 bp/SNP, ranging from 176.41 bp/SNP on Chromosome 7 to 111.73 bp/SNP on Chromosome 11. On average, a 100 kb physical window contained approximately 722.30 high-quality SNPs, indicating a high marker density for population genetic structure analyses (Table S1).

HWE tests showed that 4,539,478 SNPs (93.09%) did not significantly deviate from HWE (*p*-value adjusted by FDR), whereas the remaining 6.91% (337,196 SNPs) of loci exhibited varying degrees of deviation (FDR < 0.05). The proportion of deviating loci was generally uniform across chromosomes, with almost all chromosomes showing 4–9% of loci out of equilibrium. However, Chromosome 17 exhibited a higher deviation, with over 13.31% of SNPs departing from HWE (Table S2). Overall, almost all SNPs were under HWE, indicating that the sampled population was essentially under random mating (Figure 2B).

Estimates of inbreeding coefficients (*F_is_*) were performed using 4,876,674 high-quality SNPs among 66 individuals. The results showed that the mean observed heterozygosity (*H_o_*) was 0.3042 ± 0.000064, while the mean expected heterozygosity (*H_e_*) was 0.3241 ± 0.000056. The mean inbreeding coefficient (*F_is_*) was 0.0614 (95% CI: [0.0612, 0.0617]) (Figure 2C). The *F_is_* results indicated that the sampled population was predominantly outcrossing, with low levels of selfing or inbreeding.

Genome-wide nucleotide diversity (π) was assessed using a 10 kb sliding window with a 1 kb step. Chromosome 4 exhibited the highest mean nucleotide diversity ($\bar{\pi }$ = 0.0029), followed by chromosome 11 ($\bar{\pi }$ = 0.0028); whereas chromosome 7 had the lowest diversity level ($\bar{\pi }$ = 0.0017). The highest window-based π value was on chromosome 19 (maximum π = 0.0138), indicating local enrichment of genetic variation (Table S3). The genome-wide mean $\bar{\pi }$ was 0.0024 ± 0.0003, and the π values showed a pronounced right-skewed distribution (skewness = 0.8935). This suggested that most genomic regions exhibited low levels of variation, while a few regions displayed elevated polymorphism (Figure 2D,E). Overall, the population exhibited relatively high genetic variation among sites, and this heterogeneous distribution was likely associated with local differences in recombination rates, natural selection, or genomic structural features.

LD decayed rapidly with an increase in physical distance. At the shortest distance interval, the average r^2^ was 0.5567, and LD decreased by half (0.2784) at approximately 2.36 kb. When the distance increased to 275.47 kb, r^2^ dropped to a low level (below 0.1) (Figure 2F). The results indicated that the genome of *N. cadamba* exhibited fast LD decay, suggesting a high level of recombination rate, a feature consistent with an outcrossing mating system.

After strict quality control and LD pruning of SNP data, a total of 345,349 high-quality and largely independent SNPs were retained for population structure analysis. GRM among 66 individuals was calculated using 345,349 SNPs. With these SNPs, we obtained 66 eigenvalues (λ_i_, i = 1, …, 66), and their contributions are detailed in Table S4. The first two eigenvalues, $\lambda_{1}$ = 3.52 and $\lambda_{2}$ = 2.89, were greater than 1.0, and their principal components explained 5.51% and 4.52% of the total variation, respectively. We plotted Figure 2G with the first two principal components since they jointly explained the greatest proportion of variation (10.03%) compared to any other two principal components. Figure 2G showed a clear tendency toward family-level clustering. The clusters of 66 seedlings of 11 families were consistent with the expectation of half-sib family structure. Samples within each family shared a common maternal parent but were separated among families. The substantial intra-family variation arose from different paternal contributions, and each half-sib progeny could receive different paternal alleles through random fertilization by diverse male parents, which reflected a potential outcrossing system. Notably, Family 7 formed a relatively closer cluster separated from the other families, indicating a relatively narrow genetic variation within this family. Overall, population genetic variation was primarily distributed within families, consistent with the genetic background of half-sib families, and suggested that the species is predominantly outcrossing.

### 3.2. Mating System Analysis

#### 3.2.1. Independent SNP Filtering

We used VCFtools to remove 58,328 SNP loci that significantly deviated from HWE (*p* < 10^−6^). Subsequently, strict LD pruning was performed using PLINK (--indep-pairwise 50 10 0.1), resulting in 175,609 approximately independent SNPs for analysis with MLTR and BORICE. Among these, Chromosome 1 had the most independent SNPs (11,180), while Chromosome 17 had the fewest SNPs (6031). Chromosome 9 contained the highest proportion of independent SNPs (8682) relative to its total SNPs, about 4.14%, while Chromosome 16 had the lowest proportion (6524), about 2.98% (Table S5).

#### 3.2.2. Estimation with MLTR

**Figure 3:**
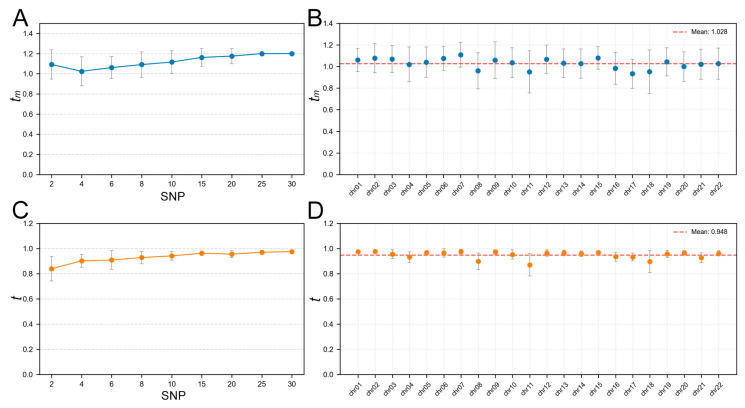
Estimation of outcrossing rates using MLTR and BORICE: the trends across SNP gradients and chromosomes. (**A**) Trend of *t_m_* estimated by MLTR in different SNP numbers. (**B**) Scatter distribution of *t_m_* values estimated by MLTR with 6 SNPs randomly sampled on each chromosome. (**C**) Trend of *t* values estimated by BORICE with different SNP numbers. (**D**) Scatter distribution of *t* values estimated by BORICE with 30 SNPs on each chromosome. The dots in blue in (**A**) and in orange in (**C**) are the mean estimates from 30 simulations under different numbers of independent SNPs. The dots in blue in (**B**) and in orange in (**D**) are the means of independent estimates with randomly sampled 6 and 30 independent SNPs on each chromosome, respectively. The error bars on each dot represent one standard deviation of estimates.

We first performed simulations based on a gradient of SNP numbers (2, 4, 6, 8, 10, 15, 20, 25, 30) to screen for an optimal number of SNPs. The results showed that *t_m_* estimates decreased from 1.092 ± 0.145 to 1.024 ± 0.144 when two and four SNP loci were used, respectively. For 6 SNP loci, the *t_m_* value was 1.061 with the SD declining to 0.108. When the number of SNPs increased to 8, *t_m_* estimates slightly increased to 1.091, accompanied by an increased SD of 0.126. From 10 to 25 SNPs, estimated *t_m_* exhibited a gradual upward trend. At 25 and 30 SNPs, *t_m_* estimates reached the upper threshold of the MLTR algorithm, with *t_m_* being fixed at 1.20 and SD reduced to 0, rendering these datasets statistically meaningless. Considering that too few SNP loci lead to substantial sampling variability, whereas an excess of SNPs skewed *t_m_* estimates toward the meaningless algorithm threshold, we selected 6 SNPs as the optimal compromise. At this gradient, the *t_m_* was 1.061 with a relatively mild bias and a low SD of 0.108. This configuration avoided the elevated data variability seen at 8 SNP loci, as well as the boundary saturation occurring at higher SNP quantities (Figure 3A; Table S6). Therefore, 6 SNPs were selected for subsequent mating system analyses.

Based on the above screening strategy, 6 SNP loci were randomly sampled per chromosome, with the sampling procedure independently repeated 30 times for each chromosome. The results revealed that the *t_m_* was estimated at 1.028 ± 0.048 (Figure 3B), while the *t_s_* was 1.015 ± 0.060 (Figure S1; Table S7). Both *t_m_* and *t_s_* estimates indicated that this population exhibited complete outcrossing.

**Figure 4:**
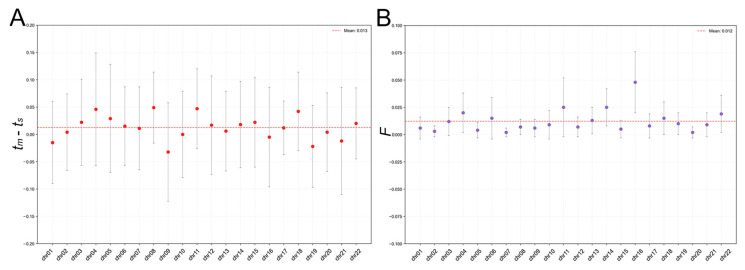
Distribution of mating system parameters in *N. cadamba* (175,609 SNPs). (**A**) Distribution of *t_m_* − *t_s_* estimates by MLTR across 22 chromosomes; (**B**) distribution of *F* estimates by BORICE across 22 chromosomes.

The difference *t_m_* − *t_s_* was estimated to be 0.013 ± 0.022 (Figure 4A; Table S7), indicating the absence of significant biparental inbreeding, and outcrossing events mainly occurring between unrelated individuals.

**Figure 5:**
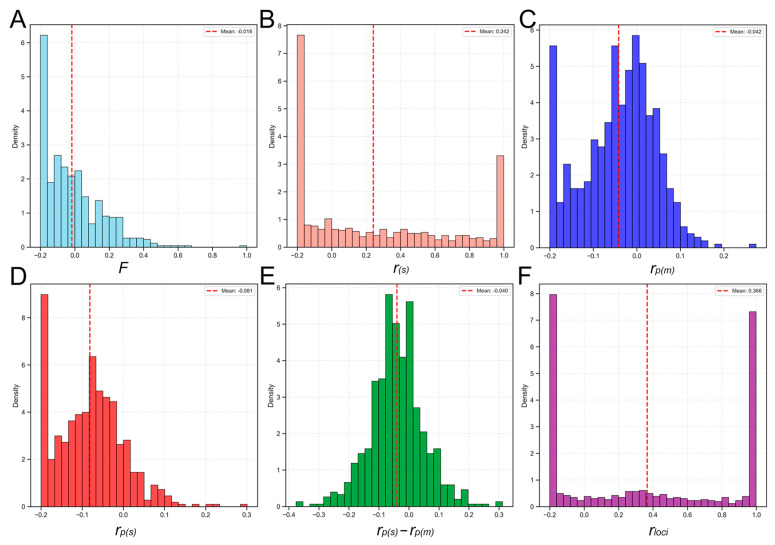
Distribution of additional mating system parameters estimated by MLTR (175,609 SNPs). (**A**) Distribution of *F* estimates; (**B**) distribution of *r*_(*s*)_ estimates; (**C**) distribution of *r_p_*_(*m*)_ estimates; (**D**) distribution of *r_p_*_(*s*)_ estimates; (**E**) distribution of the difference, *r_p_*_(*s*)_ − rp(m), estimates; (**F**) distribution of r(loci) estimates.

Maternal inbreeding coefficient (*F*) was −0.018 ± 0.171, which was not significantly different from zero, indicating that the maternal population underwent random mating without inbreeding. The among-family selfing correlation (*r_s_*) was not significantly different from zero (0.242 ± 0.440), suggesting that mating patterns varied greatly among families. The multilocus correlation of paternity (*r_p_*_(*m*)_) was −0.042 ± 0.084, showing that the pollen donors were highly diverse. The single-locus correlation of paternity (*r_p_*_(*s*)_) was −0.081 ± 0.081, which was consistent with *r_p_*_(*m*)_ and further confirmed the extensive diversity of pollen sources. The difference (*r_p_*_(*s*)_ − *r_p_*_(*m*)_) was −0.040 ± 0.090, which demonstrated no obvious genetic substructure within the studied population. The among-locus selfing correlation (*r_loci_*) was 0.366 ± 0.505, which indicated that the correlation was not significant from zero since the large standard deviation indicated statistically non-significance from zero (Table 2, Figure 5A–F).

### 3.3. Estimation with BORICE

Analysis with BORICE revealed that, as the SNP number increased from 2 to 30, the estimated outcrossing rate increased gradually, with the mean *t* values ranging from 0.839 ± 0.097 (2 SNPs) to 0.975 ± 0.009 (30 SNPs) (Table S8). Meanwhile, the SD of *t* values decreased markedly and stabilized (Figure 3C). Based on the convergence trend of *t* values and stable estimation accuracy observed above, 30 SNPs were selected for subsequent analyses.

Based on the above strategy, we randomly selected 30 SNPs per chromosome with 30 independent sampling replicates for calculation. The results indicated that the estimated population *t* value was 0.948 ± 0.029 (Figure 3D), and the estimated *F* value was 0.012 ± 0.010 (Figure 4B, Table S9). BORICE analysis indicated that the population underwent random outcrossing with negligible biparental inbreeding.

## 4. Discussion

### 4.1. Mating System

This study systematically estimated key parameters of the mating system using two different methods, MLTR and BORICE, based on genome-wide SNP markers in a natural population of *N. cadamba*. Both methods were basically consistent in quantifying the mating system and showed an essentially outcrossing system. The MLTR analysis showed that *t_m_* was 1.028 ± 0.048 and *t_s_* was 1.015 ± 0.060 (Table 2), which was not significantly different from 1. Note that the MLTR method used the Newton–Raphson iterative approach in computation, which did not impose [0, 1] boundary constraints on the parameters; therefore, under finite sample conditions, random sampling error could cause the estimates to slightly exceed the theoretical upper limit [28]. Similar phenomena were also reported in other plant populations, such as in palms and *Toona ciliata* [25,37], indicating complete outcrossing or an extremely high level of outcrossing. The BORICE analysis indicated that t was 0.948 ± 0.029, slightly lower than the *t_m_* estimate from MLTR (0.948 vs. 1.028). Such differences mainly resulted from the difference in statistical algorithms. The BORICE analysis used Bayesian priors and reflection boundaries to effectively constrain the parameters within [0, 1], yielding more conservative and robust estimates under small sample conditions [29].

Regarding biparental inbreeding, the *t_m_* − *t_s_* value was 0.013 ± 0.022, and no significant biparental inbreeding was detected in the population, indicating that outcrossing mainly occurred between unrelated individuals. Meanwhile, the *F* estimates from the BORICE analysis (0.012 ± 0.010) or from MLTR (−0.018 ± 0.171) suggested that the maternal individuals themselves had no obvious inbreeding. With respect to paternal correlation, *r_p_*_(*m*)_ (−0.042 ± 0.084) and *r_p_*_(*s*)_ (−0.081 ± 0.081) were close to zero, indicating that the probability of progeny sharing the same father was extremely low and the effective pollen sources were highly diverse. The *r_p_*_(*s*)_ − *r_p_*_(*m*)_ difference (−0.040 ± 0.090) was close to zero, suggesting that there was no obvious genetic substructure in the population and mating was not biased by population stratification.

Nevertheless, the HWE test showed that 6.91% of all SNP loci deviated significantly from HWE, and the estimated $F_{is}$ was 0.0614 (Figure 2B,C). This reflected the feature of the investigated samples, where inbreeding occurred among half-sibs within each family. Given that both MLTR and BORICE analyses indicated that biparental inbreeding was negligible, such deviations were likely not driven by inbreeding within the mating system, but primarily by the presence of half-sib families in the investigated samples.

PCA detected distinct clustering patterns at the family level (Figure 2G). PCA was performed using 345,349 high-quality and largely independent SNPs, resulting in an extremely high-dimensional dataset with genetic information broadly distributed across numerous loci. When genetic variance is distributed across many principal components, the first two components can only capture the most prominent directions of variation. The samples used in this study were derived from half-sib families within a single natural population, where family relationships were well-defined and population structure was relatively simple. Under this situation, there was no single “dominant” factor driving the overall genetic variation, and the variation tended to be dispersed across multiple independent axes, preventing the first two principal components from concentrating a large proportion of the total variance. Nevertheless, the first two components (PC1 and PC2) successfully distinguished the half-sib family structure and variation within half-sib families, which implies the feature of the outcrossing system.

Individuals within each family shared the same maternal parent and exhibited varying degrees of genetic relatedness, which reflected the impacts of population structure, particularly the Wahlund effect [16,38]. When different families were pooled for joint analysis, differences in allele frequencies among families triggered the Wahlund effect. This phenomenon led to homozygote excess in the overall samples, thereby causing deviations from HWE and inflated $F_{is}$ estimates, rather than reflecting the inbreeding level of the parental population.

The LD decay analysis provided further corroboration at the genomic level. A rapid LD decay (Figure 2F) was often associated with a high recombination frequency and large effective population size, which represented a typical hallmark of outcrossing species. For example, in outcrossing forest trees, the r^2^ half-decay distance is less than 500 bp in poplars and ranges from 500 to 2000 bp in pines [39]. By contrast, a typical selfing species, such as *Arabidopsis thaliana*, usually exhibits a much longer half-decay distance exceeding 250 kb [40]. The rapid LD decay pattern was consistent with the conclusion of the outcrossing pattern derived from MLTR and BORICE analyses in *N. cadamba*.

The mating system is highly consistent with the floral structure and pollination biology characteristics of *N. cadamba*. Zu [41] conducted observations on the floral organ development of *N. cadamba* and reported that the species exhibited typical dichogamy (specifically, protogyny) and dynamic herkogamy. The stigma became receptive 24 h before anthesis, and the peak of pollen viability occurred on the first day of anthesis, after which it declined rapidly. The receptive surface of the stigma faced away from the direction of anther dehiscence, which effectively reduced the probability of selfing and mechanically ensured outcrossing. Meanwhile, cross-pollen had a clear competitive advantage over self-pollen (whose viability had already declined significantly), and this spatiotemporal isolation reflected a preference for outcrossing in the reproductive strategy of *N. cadamba*. Furthermore, the capitulum and the yellow funnel-shaped corolla of *N. cadamba* provided visual signals for insect visitation, and the olive-shaped, rough-surfaced pollen grains were conducive to adhesion to the body surfaces of pollinators, thereby facilitating long-distance pollen dispersal. By integrating analyses of genomics, population genetics, and phenotypic observations, this study strongly supported the conclusion that the mating system of *N. cadamba* in natural populations was very likely outcrossing.

### 4.2. Implications

Note that the present study only investigated a single natural population, and more population analyses are needed to derive a more general conclusion. Given our present findings, potentially distinct mating systems between *N. cadamba* and *N. macrophylla* could yield several implications. The first implication is concerned with the relationship between mating system and a species’ range. In plant evolutionary biology, this relationship has long been appreciated but remains controversial. Grossenbacher [42], based on a phylogenetic comparative analysis of 194 sister-species pairs from 20 genera, proposed that species with autonomous selfing ability typically had geographic ranges 1.5–2 times larger than those of their outcrossing relatives. The key mechanism was that selfing provided reproductive assurance, allowing a single individual to establish a population. This prediction is likely discordant with species in the genus *Neolamarckia*. The present study found that *N. cadamba*, which was more likely to exhibit nearly obligate outcrossing, was a widespread species, whereas *N. macrophylla*, which possessed a certain selfing ability (1 − t = 23.1%), was a narrow-range species. Selfing enhances the accumulation of deleterious genes and restricts species spatial distribution [43].

The second implication is about the relative genome sizes between species in the genus *Neolamarckia*. *N. cadamba* is expected to have a larger genome size than *N. macrophylla*. Although the evolutionary divergence between *N. cadamba* and *N. macrophylla* occurred approximately 6.757 mya during the late Miocene [5], their likely distinct mating systems could shape genome size and structure [44]. A comparison of mitochondrial genomes revealed that *N. cadamba* possessed a larger mitochondrial genome (414,980 bp vs. 289,793 bp) and more functional genes (83 vs. 58), indicating a more complete genetic basis for energy metabolism and environmental stress responses. A comparison of nuclear genome size awaits data collection.

The third implication is that the present finding supports previous findings of population genetic differentiation of *N. cadamba* in China, since the mating system affects gene flow between populations [45]. A previous study using maternally inherited mtDNA and biparentally inherited nrDNA ITS markers indicated that in natural populations of *N. cadamba*, pollen flow was much greater than seed flow (their ratio >> 1.0). Population differentiation with nrDNA ITS markers was extremely low (*F_st_* ≈ 3%), and no significant isolation by distance (IBD) was detected [14]. *N. cadamba* did not require selfing for reproductive assurance; relying on its extremely strong long-distance pollen flow, it overcame mate limitation, which laid the foundation for its wide distribution in southern China, Southeast Asia, South Asia, and other regions.

In contrast, *N. macrophylla* was naturally distributed on Sulawesi Island and the Maluku Islands of Indonesia, where its habitats were fragmented and spatial isolation was pronounced. Paternity analysis based on SSR markers indicated that its pollen flow exhibited a clear distance-decay pattern; although the maximum distance reached 339 m, the vast majority of effective pollination events and pollen contributions were tightly restricted to short distances of 0–161 m [13]. Spatial isolation and limitations of local pollinators together constitute barriers to pollen movement, which might have promoted the evolution of a certain proportion of selfing in *N. macrophylla*, resulting in a mixed mating system. In addition, its mitochondrial genome experienced significant gene loss (e.g., cox2) and a reduction in non-coding regions. This genome degradation was possibly related to its long-term persistence in narrow, fragmented habitats, limited effective population size, and inbreeding depression, and it might also have been a factor leading to reduced environmental adaptability and restricted distribution.

The comparison of the mating systems of the two closely related *Neolamarckia* species suggested that, in woody plants, an efficient pollen dispersal mechanism could effectively substitute for the reproductive assurance provided by selfing, enabling outcrossing species to achieve a wide distribution. The size of a species’ distribution range might be influenced not only by the mating system, but also by a multi-dimensional systematic interplay of factors such as life-history strategy, pollinator efficiency, and gene flow intensity.

## 5. Conclusions

With genome-wide SNPs, this study showed that *N. cadamba* was essentially outcrossing in a natural population. No significant biparental inbreeding was detected, and the maternal inbreeding coefficient was nearly zero, indicating that the population relied on random outcrossing. The paternal correlations *r_p_*_(*m*)_ and *r_p_*_(*s*)_ were close to zero, indicating a highly diverse pool of effective pollen donors and an extremely low probability of progeny sharing the same paternal parent, thereby maintaining a high level of genetic diversity within the population. This study provided evidence that *N. cadamba* could have a distinct mating system from *N. macrophylla*, which implies its multiple important effects on ecology, evolutionary consequences, and breeding.

## Figures and Tables

**Table 1:** Eleven half-sib families and samples for genome resequencing in the Jinghong population of *N. cadamba*.

Half-Sib Family ID	Sample ID *
950	C76, C77, C80, C81, D83, D84
946	C41, C61, C62, C79, D81, D82
945	C56, C57, C71, C72, D79, D80
931	C7, C48, C49, C74, D71, D72
928	C45, C68, C69, C70, D69, D70
924	C36, C37, C51, C52, D67, D68
922	C21, C53, C54, C55, D65, D66
919	C3, C4, C63, C64, D61, D62
917	C5, C6, C42, C58, D59, D60
914	C8, C9, C10, C25, D55, D56
913	C44, C65, C66, C67, D53, D54

*: C-series samples came from CNGB with accession number CNP0007971 and D-series samples were re-sequenced in Zhao et al. [15].

**Table 2:** The mating system parameters estimated by MLTR.

Parameters	Mean	SD
*t_m_*	1.028	0.048
*t_s_*	1.015	0.060
*t_m_ − t_s_*	0.013	0.022
*F* (maternal)	−0.018	0.171
*r_s_*	0.242	0.440
*r_p_*_(*m*)_	−0.042	0.084
*r_p_*_(*s*)_	−0.081	0.081
*r_p_*_(*s*)_ − *r_p_*_(*m*)_	−0.040	0.090
*r_loci_*	0.366	0.505

## Data Availability

All data used in this article were included in the main text.

## Supplementary Materials

The following supporting information can be downloaded at: https://www.mdpi.com/article/10.3390/biology15151262/s1, File S1: Genome resequencing of *N. cadamba*. References [46,47,48,49,50,51,52] are cited in the Supplementary Materials. Figure S1: Distribution of *t_s_* estimates obtained by MLTR across 22 chromosomes in *N. cadamba*. Table S1: Distribution of SNP sites across 22 chromosomes of *N. cadamba*; Table S2: Distribution of SNP sites deviating from Hardy–Weinberg equilibrium across chromosomes of *N. cadamba*; Table S3: Distribution of nucleotide diversity (π) across chromosomes of *N. cadamba*; Table S4: The eigenvalues and explained variances of 66 principal components in *N. cadamba*; Table S5: Independent SNPs retained after HWE and LD pruning across *N. cadamba*; Table S6: The multilocus outcrossing rate (*t_m_*) estimated by MLTR with different numbers of SNP sites; Table S7: Estimates of *t_m_*, *t_s_* and *t_m_* − *t_s_* values by MLTR with 6 SNPs across chromosomes. Table S8: The *t* estimate by BORICE under different numbers of SNP sites. Table S9: Estimates of *t* and *F* by BORICE with 30 SNPs across chromosomes.
