# Supplementary material for: Scoring Mating System of a Natural Population of Neolamarckia cadamba with Genome Resequencing Data
Source: Biology (Basel). 2026 Aug 1;15(15):1262. doi: 10.3390/biology15151262 (PMC13465438; doi:10.3390/biology15151262)
Supplement: Supplementary file 1 [file biology-15-01262-s001.zip › biology-4436935-supplementary.pdf]

Supporting information

Scoring mating system of a natural population of *Neolamarckia cadamba* with genome resequencing data

1. Genome resequencing of *N. cadamba*;
2. Figure S1
3. Tables S1-S9

File S1. Genome resequencing of *N. cadamba*

The experimental procedure followed the standard protocol provided by Illumina, including DNA sample quality check, library construction, library quality testing, and library sequencing. Genomic DNA was extracted using the cetyltrimethylammonium bromide (CTAB) method [46]. The concentration and purity of gDNA were determined using a Nanodrop 2000 spectrophotometer and a Qubit 3.0 fluorometer (Thermo Fisher Scientific, Massachusetts USA). DNA integrity was evaluated using 1.0% agarose gel. The qualified DNA samples had a concentration of more than 5 ng /μl and the total amount was not less than 0.5 μg, with the main diffusion bands of more than 6 kb in size for integrity and no obvious impurities.

After sample quality control (QC), a total amount of 0.2 μg DNA per sample was used as input material for the DNA library preparations. Briefly, genomic DNA sample was fragmented by sonication to a size of 350 bp. Then DNA fragments were endpolished, A-tailed, and ligated with the full-length adapter for Illumina sequencing, followed by further size selection and PCR amplification. After PCR products were purified using the AMPure XP system (Beverly USA). Subsequently, library quality was assessed on the Agilent 5400 system (Agilent, USA) and quantified by qPCR (1.5 nM).

The qualified libraries were pooled and sequenced on Illumina platforms using PE150 strategy, according to effective library concentration and data amount required.

The raw data from the Illumina platform were transformed and recorded in FASTQ format [47]. Sequence artifacts, including reads containing adapter contamination, low quality nucleotides and unrecognizable nucleotides (N), were controlled by following data processing:
(1) Discard a paired of reads if either one read contains adapter contamination (>10 nucleotides aligned to the adapter, allowing ≤ 10% mismatches);
(2) Discard a paired of reads if more than 10% of bases are uncertain in either one read;
(3) Discard a paired of reads if the proportion of low quality (Phred quality <5) bases is over 50% in either one read.

Reads were mapped to the reference genome using the Burrows Wheeler Aligner (BWA) software to get the original mapping results stored in BAM format (parameter: mem -t 4 -k 32 -M) [48]. Then, the results were dislodged duplication by SAMtools (parameter: rmdup) and Picard (http://broadinstitute.github.io/picard/) [49].

The raw SNP/InDel sets were called by SAMtools with the parameters as '-C 50 - mpileup -m2 -F 0.002 -d 1000'. Then we filtered these sets using the following criteria: (1) The depth of the variate position >4 and (2) The mapping quality >20.

CNVs were detected by CNVnator to acquire potential deletion and duplication (parameter: -call 100) [50]. SVs were detected by BreakDancer [51]. ANNOVAR was used for functional annotation of variants [52]. The UCSC known genes were used for gene and region annotations.

References:

46. Doyle, J.J.; Doyle, J.L. A rapid DNA isolation procedure for small quantities of fresh leaf tissues. *Phytochem Bull* **1987**, *19*, 11–15.
47. Cock, P.J.; Fields, C.J.; Goto, N.; Heuer, M.L.; Rice, P.M. The Sanger FASTQ file format for sequences with quality scores, and the Solexa/Illumina FASTQ variants. *Nucleic Acids Res.* **2010**, *38(6)*, 1767-1771. https://doi.org/10.1093/nar/gkp1137
48. Li, H.; Durbin, R. Fast and accurate short read alignment with Burrows-Wheeler transform. *Bioinformatics* **2009**, *25*, 1754-1760. https://doi.org/10.1093/bioinformatics/btp324
49. Li, H.; Handsaker, B.; Wysoker, A.; Fennell, T.; Ruan, J.; Homer, N.; Marth, G.; Abecasis, G.; Durbin, R.; 1000 Genome Project Data Processing Subgroup. The Sequence Alignment/Map format and SAMtools. *Bioinformatics* **2009**, *25(16)*, 2078–2079. https://doi.org/10.1093/bioinformatics/btp352
50. Abyzov, A.; Urban, A.E.; Snyder. M.; Gerstein, M. CNVnator: an approach to discover, genotype, and characterize typical and atypical CNVs from family and population genome sequencing. *Genome Res.* **2011**, *21(6)*, 974-984. https://doi.org/10.1101/gr.114876.110
51. Chen, K.; Wallis, J.W.; McLellan, M.D.; Larson, D.E.; Kalicki, J.M.; Pohl, C.S.; McGrath, S.D.; Wendl, M.C.; Zhang, Q.; Locke, D.P.; et al. BreakDancer: an algorithm for high-resolution mapping of genomic structural variation. *Nat Methods.* **2009**, *6(9)*, 677–681. https://doi.org/10.1038/nmeth.1363
52. Wang, K.; Li, M.; Hakonarson, H. ANNOVAR: functional annotation of genetic variants from high-throughput sequencing data. *Nucleic Acids Res.* **2010**, *38(16)*, e164.
https:// doi.org/10.1093/nar/gkq603

Figure S1. Distribution of t_s estimates obtained by MLTR across 22 chromosomes in *N. cadamba*

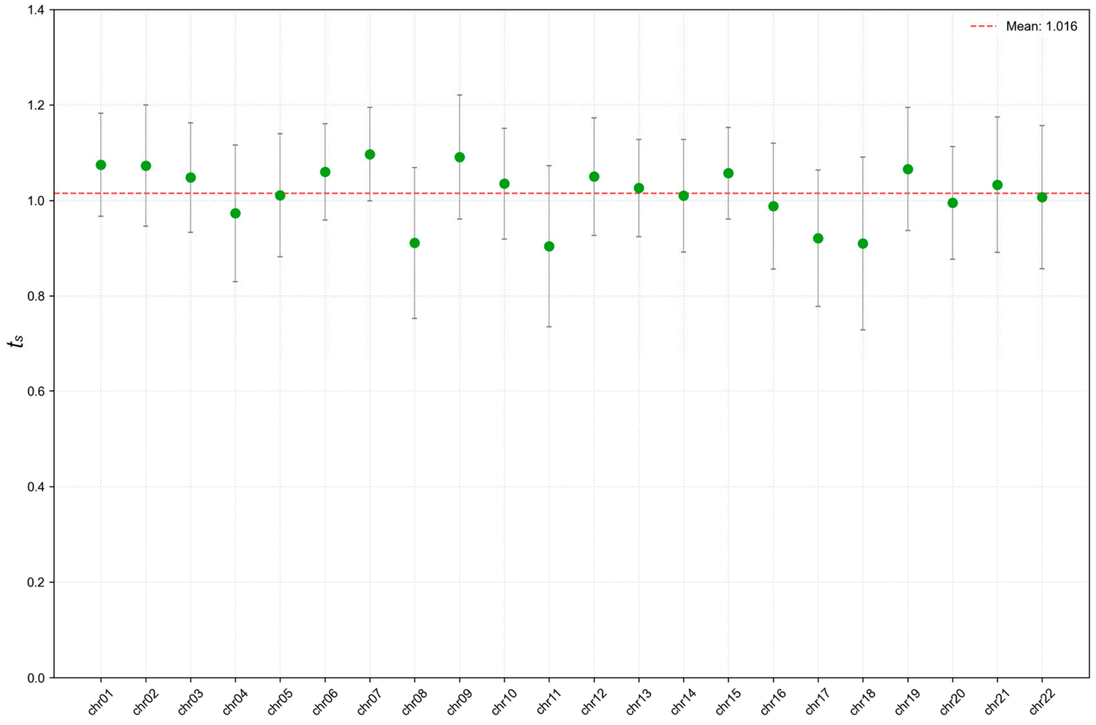

Table S1. Distribution of SNP sites across 22 chromosomes of *N. cadamba*

Chromosome	Total SNP	Min position	Max position	Density (bp / SNP)	SNPs /100kb	Percentage(%)
chr01	321,784	3,365	40,167,398	124.82	801.2	6.60
chr02	240,712	9,382	36,798,086	152.83	654.3	4.94
chr03	279,440	13,383	35,147,831	125.73	795.4	5.73
chr04	283,890	14,166	34,713,012	122.23	818.1	5.82
chr05	239,061	2,498	34,116,030	142.70	700.8	4.90
chr06	220,966	928	33,796,703	152.95	653.8	4.53
chr07	190,885	1,562	33,675,061	176.41	566.9	3.91
chr08	250,865	308	32,439,964	129.31	773.3	5.14
chr09	209,850	389	31,407,371	149.66	668.2	4.30
chr10	195,473	342	29,334,823	150.07	666.4	4.01
chr11	261,285	3,279	29,196,210	111.73	895.0	5.36
chr12	209,282	2,486	29,144,404	139.25	718.1	4.29
chr13	194,691	12,893	28,929,231	148.52	673.3	3.99
chr14	217,797	33,783	28,548,636	130.92	763.8	4.47
chr15	222,379	466	27,537,281	123.83	807.6	4.56
chr16	219,201	13,980	27,217,201	124.10	805.8	4.49
chr17	184,121	17,907	27,041,525	146.77	681.3	3.78
chr18	205,645	259	27,025,816	131.42	760.9	4.22
chr19	173,766	796	26,396,942	151.91	658.3	3.56
chr20	185,020	22,710	25,678,496	138.66	721.2	3.79
chr21	167,785	894	25,371,675	151.21	661.3	3.44
chr22	202,776	3,542	24,501,539	120.81	827.7	4.16
Overall	4,876,674	-	-	138.45	722.3	100

Table S2. Distribution of SNP sites deviating from Hardy–Weinberg equilibrium across chromosomes of *N. cadamba*

Chromosome	FDR< 0.05	Total SNPs of Chromosome	Percentage
chr01	18,789	321,784	5.84%
chr02	11,925	240,712	4.95%
chr03	16,433	279,440	5.88%
chr04	21,338	283,890	7.52%
chr05	15,636	239,061	6.54%
chr06	14,542	220,966	6.58%
chr07	11,304	190,885	5.92%
chr08	19,889	250,865	7.93%
chr09	16,955	209,850	8.08%
chr10	17,003	195,473	8.70%
chr11	14,762	261,285	5.65%
chr12	15,009	209,282	7.17%
chr13	8,113	194,691	4.17%
chr14	11,683	217,797	5.36%
chr15	9,460	222,379	4.25%
chr16	15,307	219,201	6.98%
chr17	24,514	184,121	13.31%
chr18	18,196	205,645	8.85%
chr19	16,149	173,766	9.29%
chr20	12,130	185,020	6.56%
chr21	13,168	167,785	7.85%
chr22	14,891	202,776	7.34%
Overall	337,196	4,876,674	6.91%

Table S3. Distribution of nucleotide diversity (π) across chromosomes of *N. cadamba*

Chromosome	Mean π	SD	Median	Min	Max	Range
chr01	0.0025	0.0015	0.0025	0.0000	0.0115	0.0115
chr02	0.0020	0.0015	0.0017	0.0000	0.0095	0.0095
chr03	0.0026	0.0016	0.0024	0.0000	0.0132	0.0132
chr04	0.0029	0.0019	0.0027	0.0000	0.0129	0.0129
chr05	0.0022	0.0014	0.0018	0.0000	0.0118	0.0118
chr06	0.0020	0.0014	0.0019	0.0000	0.0132	0.0132
chr07	0.0017	0.0014	0.0013	0.0000	0.0101	0.0100
chr08	0.0025	0.0015	0.0024	0.0000	0.0097	0.0097
chr09	0.0023	0.0015	0.0021	0.0000	0.0134	0.0134
chr10	0.0021	0.0014	0.0019	0.0000	0.0103	0.0103
chr11	0.0028	0.0018	0.0027	0.0000	0.0122	0.0122
chr12	0.0024	0.0016	0.0022	0.0000	0.0113	0.0113
chr13	0.0021	0.0014	0.0018	0.0000	0.0085	0.0085
chr14	0.0024	0.0016	0.0021	0.0000	0.0125	0.0125
chr15	0.0025	0.0014	0.0023	0.0000	0.0100	0.0100
chr16	0.0028	0.0016	0.0027	0.0000	0.0111	0.0111
chr17	0.0024	0.0017	0.0022	0.0000	0.0105	0.0105
chr18	0.0023	0.0015	0.0022	0.0000	0.0103	0.0103
chr19	0.0022	0.0016	0.0018	0.0000	0.0138	0.0138
chr20	0.0023	0.0015	0.0022	0.0000	0.0106	0.0106
chr21	0.0020	0.0015	0.0016	0.0000	0.0104	0.0104
chr22	0.0028	0.0018	0.0028	0.0000	0.0121	0.0121
Overall	0.0024	0.0003	-	-	-	-

Table S4. The eigenvalues and explained variances of 66 principal components in *N. cadamba*

Principal Component	Eigenvalue	Principal Component (Variance Percentage)	Cumulative Contribution Rate (%)
PC1	3.5179	5.5124	5.5124
PC2	2.8861	4.5223	10.0347
PC3	2.5677	4.0235	14.0583
PC4	2.2714	3.5591	17.6173
PC5	2.1621	3.3879	21.0052
PC6	2.0611	3.2296	24.2348
PC7	1.9357	3.0331	27.2679
PC8	1.8223	2.8555	30.1233
PC9	1.8049	2.8281	32.9514
PC10	1.7284	2.7083	35.6597
PC11	1.5389	2.4113	38.0711
PC12	1.4537	2.2778	40.3489
PC13	1.4098	2.2091	42.5579
PC14	1.3320	2.0871	44.6450
PC15	1.2462	1.9527	46.5977
PC16	1.1569	1.8128	48.4105
PC17	1.1238	1.7609	50.1715
PC18	1.0517	1.6479	51.8194
PC19	1.0235	1.6038	53.4231
PC20	1.0030	1.5717	54.9949
PC21	0.9970	1.5623	56.5572
PC22	0.9401	1.4732	58.0303
PC23	0.9200	1.4416	59.4719
PC24	0.9002	1.4106	60.8825
PC25	0.8919	1.3976	62.2801
PC26	0.8528	1.3363	63.6164
PC27	0.8341	1.3070	64.9235
PC28	0.8184	1.2823	66.2058
PC29	0.8144	1.2761	67.4819
PC30	0.7965	1.2480	68.7299
PC31	0.7845	1.2292	69.9591
PC32	0.7764	1.2165	71.1756
PC33	0.7581	1.1879	72.3635
PC34	0.7427	1.1638	73.5273
PC35	0.7380	1.1564	74.6837
PC36	0.7214	1.1304	75.8141
PC37	0.7181	1.1252	76.9393
PC38	0.7114	1.1148	78.0541

PC39	0.7069	1.1077	79.1618
PC40	0.6868	1.0762	80.2380
PC41	0.6816	1.0680	81.3060
PC42	0.6719	1.0529	82.3589
PC43	0.6626	1.0382	83.3971
PC44	0.6523	1.0221	84.4192
PC45	0.6355	0.9957	85.4149
PC46	0.6160	0.9652	86.3801
PC47	0.6117	0.9585	87.3386
PC48	0.5848	0.9163	88.2549
PC49	0.5754	0.9017	89.1565
PC50	0.5726	0.8972	90.0537
PC51	0.5579	0.8743	90.9280
PC52	0.5450	0.8539	91.7819
PC53	0.5364	0.8405	92.6224
PC54	0.5259	0.8240	93.4464
PC55	0.5156	0.8079	94.2543
PC56	0.5092	0.7979	95.0522
PC57	0.4974	0.7794	95.8316
PC58	0.4865	0.7623	96.5940
PC59	0.4752	0.7447	97.3386
PC60	0.4665	0.7310	98.0696
PC61	0.4226	0.6622	98.7318
PC62	0.2364	0.3705	99.1023
PC63	0.2129	0.3336	99.4359
PC64	0.2112	0.3310	99.7669
PC65	0.1877	0.2941	100.0611
PC66	-0.0390	-0.0611	100.0000

Table S5. Independent SNPs retained after HWE and LD pruning across *N. cadamba*

Chromosome	Total SNPs of Chromosome	Post-HWE SNPs	Independent SNPs	Remained SNPs (%)
chr01	321,784	318,595	11180	3.47
chr02	240,712	238,204	9331	3.88
chr03	279,440	276,790	9567	3.42
chr04	283,890	279,351	8628	3.04
chr05	239,061	236,365	8541	3.57
chr06	220,966	217,486	8472	3.83
chr07	190,885	188,165	7701	4.03
chr08	250,865	249,352	8681	3.46
chr09	209,850	204,659	8682	4.14
chr10	195,473	192,544	8070	4.13
chr11	261,285	259,798	8743	3.35
chr12	209,282	207,322	7389	3.53
chr13	194,691	193,302	6815	3.50
chr14	217,797	215,507	7936	3.64
chr15	222,379	220,987	8021	3.61
chr16	219,201	216,671	6524	2.98
chr17	184,121	181,574	6031	3.28
chr18	205,645	203,302	7366	3.58
chr19	173,766	168,602	7075	4.07
chr20	185,020	182,891	7015	3.79
chr21	167,785	165,761	6936	4.13
chr22	202,776	201,118	6905	3.41
Overall	4,876,674	4,818,346	175,609	3.60%

Table S6. The multilocus outcrossing rate (t_m) estimated by MLTR with different numbers of SNP sites

SNP	t_m	SD
2	1.092	0.145
4	1.024	0.144
6	1.061	0.108
8	1.091	0.126
10	1.116	0.113
15	1.162	0.088
20	1.175	0.076
25	1.200	0.000
30	1.200	0.000

Table S7. Estimates of t_m, t_s and t_m–t_s values by MLTR with 6 SNPs across chromosomes

Chromosome	t_m	SD	t_s	SD	t_m–t_s	SD
chr01	1.061	0.108	1.075	0.108	-0.015	0.075
chr02	1.077	0.134	1.073	0.127	0.004	0.070
chr03	1.069	0.125	1.048	0.115	0.022	0.079
chr04	1.020	0.162	0.973	0.143	0.046	0.103
chr05	1.041	0.141	1.011	0.129	0.029	0.099
chr06	1.075	0.110	1.060	0.101	0.015	0.072
chr07	1.108	0.113	1.097	0.098	0.011	0.076
chr08	0.960	0.167	0.911	0.158	0.049	0.065
chr09	1.059	0.170	1.091	0.130	-0.032	0.090
chr10	1.036	0.137	1.035	0.116	0.000	0.079
chr11	0.951	0.195	0.904	0.169	0.047	0.073
chr12	1.067	0.131	1.050	0.123	0.017	0.090
chr13	1.031	0.132	1.026	0.102	0.006	0.073
chr14	1.027	0.135	1.010	0.118	0.018	0.079
chr15	1.079	0.104	1.057	0.096	0.022	0.082
chr16	0.984	0.147	0.988	0.132	-0.005	0.091
chr17	0.933	0.135	0.921	0.143	0.012	0.049
chr18	0.952	0.202	0.910	0.181	0.042	0.072
chr19	1.044	0.130	1.066	0.129	-0.022	0.075
chr20	1.000	0.136	0.995	0.118	0.004	0.072
chr21	1.021	0.140	1.033	0.142	-0.012	0.098
chr22	1.027	0.145	1.007	0.150	0.020	0.065
Overall	1.028	0.048	1.015	0.060	0.013	0.022

Table S8. The *t* estimates by BORICE under different numbers of SNP sites

SNP	*t*	SD
2	0.839	0.097
4	0.902	0.051
6	0.908	0.075
8	0.928	0.049
10	0.941	0.035
15	0.962	0.017
20	0.955	0.029
25	0.970	0.016
30	0.975	0.009

Table S9. Estimates of *t* and *F* by BORICE with 30 SNPs across chromosomes

Chromosome	*t*	SD	*F*	SD
chr01	0.975	0.009	0.006	0.010
chr02	0.976	0.010	0.003	0.005
chr03	0.955	0.034	0.012	0.013
chr04	0.931	0.042	0.020	0.018
chr05	0.967	0.015	0.004	0.007
chr06	0.965	0.033	0.015	0.019
chr07	0.975	0.019	0.002	0.004
chr08	0.898	0.066	0.007	0.007
chr09	0.972	0.019	0.006	0.008
chr10	0.952	0.036	0.009	0.013
chr11	0.870	0.088	0.025	0.027
chr12	0.964	0.025	0.007	0.009
chr13	0.965	0.022	0.013	0.012
chr14	0.958	0.021	0.025	0.017
chr15	0.967	0.016	0.005	0.008
chr16	0.935	0.035	0.048	0.028
chr17	0.932	0.031	0.008	0.011
chr18	0.896	0.088	0.015	0.015
chr19	0.957	0.028	0.010	0.010
chr20	0.965	0.018	0.002	0.005
chr21	0.926	0.039	0.009	0.011
chr22	0.961	0.021	0.019	0.017
Overall	0.948	0.029	0.012	0.010
